# Dendritic Cell Subsets in Type 1 Diabetes: Friend or Foe?

**DOI:** 10.3389/fimmu.2013.00415

**Published:** 2013-12-06

**Authors:** Penelope A. Morel

**Affiliations:** ^1^Department of Immunology, University of Pittsburgh, Pittsburgh, PA, USA

**Keywords:** dendritic cells, type 1 diabetes, T regulatory cells, autoimmunity, tolerance

## Abstract

Type 1 diabetes (T1D) is a T cell mediated autoimmune disease characterized by immune mediated destruction of the insulin-producing β cells in the islets of Langerhans. Dendritic cells (DC) have been implicated in the pathogenesis of T1D and are also used as immunotherapeutic agents. Plasmacytoid (p)DC have been shown to have both protective and pathogenic effects and a newly described merocytic DC population has been shown to break tolerance in the mouse model of T1D, the non-obese diabetic (NOD) mouse. We have used DC populations to prevent the onset of T1D in NOD mice and clinical trials of DC therapy in T1D diabetes have been initiated. In this review we will critically examine the recent published literature on the role of DC subsets in the induction and regulation of the autoimmune response in T1D.

## Introduction

**Figure 1:**
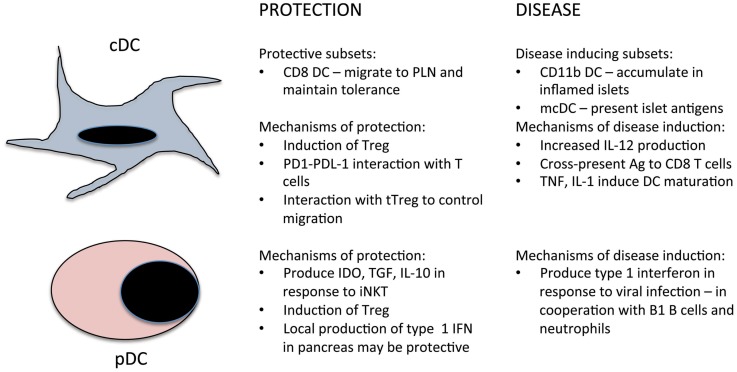
**Summary of the roles of cDC and pDC in the prevention and induction of T1D**.

Type 1 diabetes (T1D) is an autoimmune disease characterized by immune mediated destruction of the insulin-producing β cells in the islets of Langerhans of the pancreas. The pathogenesis of T1D is multifactorial with genetic, immunological, metabolic, and environmental factors all contributing (1). It begins with a loss of self-tolerance to islet-derived self-antigens, which usually occurs early in life. This could occur as a result of a viral infection targeting the pancreas or following pancreatic remodeling. These insults lead to the death of β cells, release of self-antigens, and induction of inflammatory cytokines such as TNF-α and IL-1-β. Dendritic cells (DC) present within the pancreas take up released β-cell-derived antigens and migrate to the draining lymph nodes (LN) and activate naïve islet-specific CD4 and CD8 T cells. Depending on the signals delivered to the T cell by DCs the T cells will differentiate into either inflammatory effector cells such as T helper (Th)1 cells or anti-inflammatory Th2 or regulatory (Treg) cells. Activated islet-specific T cells then migrate to the pancreas where they infiltrate and collect around the islets. The early infiltrates appear to be dominated by Th2 and Treg cells; this is termed peri-insulitis as there is little invasion into the islet. At some point, the infiltrate becomes invasive and begins to enter and destroy the β cells, and the balance between the regulatory and inflammatory T cell populations is lost. The precise factors that trigger both loss of self-tolerance and the development of invasive insulitis are not well understood. DCs play important roles at all stages of the autoimmune response in T1D due to their pivotal role in activating naïve T cells and in maintaining self-tolerance (2). This review will explore recent developments in our understanding of the roles DC play in the pathogenesis, and prevention, of T1D (Figure 1) as well as the therapeutic potential of DCs for the prevention and treatment of T1D. Much of the work in this area has been focused on the non-obese diabetic (NOD) model of the disease. This mouse strain spontaneously develops diabetes and shares many of the genetic and immunological features of the human disease (3). Where possible we will highlight similarities and differences between NOD mouse studies and relevant studies in human T1D patients.

## Dendritic Cell Subsets

Since their discovery by Ralph Steinman in the 1970s (4–6) DCs have been the subject of intense study. DCs are antigen presenting cells (APC) that bridge the innate and adaptive immune systems (7). They act as sentinels of the immune system through their location in peripheral tissues, where they form a dense network, and their ability to respond to pathogens through expression of pathogen recognition receptors such as Toll-like receptors (TLR). While in peripheral tissues DCs have an immature phenotype characterized by low levels of major histocompatibility complex (MHC) and co-stimulatory molecules (CD80, CD86) and a high endocytic rate; the function of tissue DCs is the detection and processing of antigen. Upon interaction with a pathogen DCs become activated, undergo maturation and migrate to the draining LN. This maturation leads to an increase in the expression of MHC, CD80, and CD86, a decrease in endocytosis and a change in the pattern of chemokine receptor expression. Mature DCs entering the T cell zone of the draining lymph node are primed to present antigen to naïve T cells. Even in the absence of pathogen there is a steady state migration of DC from the periphery to the LN and this is necessary for the maintenance of peripheral tolerance (8).

### DC phenotype

There are two main classes of DC: plasmacytoid DCs (pDCs) and conventional or classical DCs (cDCs) (7). pDCs are primarily located in the blood and lymphoid organs and produce high levels of type 1 interferon (IFN) following engagement of TLRs with foreign nucleic acids (9). pDCs express a restricted set of TLRs, TLR7, and TLR9, that recognize foreign RNA and DNA respectively as well as several unique markers that distinguish them from cDCs. These include BDCA-2 and ILT7 for human pDCs and SiglecH and Bst2 for murine pDCs. In the mouse pDCs are commonly identified as CD11c^low^ B220^+^ SiglecH^+^ Bst2^+^. Human pDCs are CD11c^−^ and in addition to BDCA-2 and ILT7 also express CD123 and BDCA-4 (9). There are two main populations of cDCs in mouse: CD11c^+^ CD11b^−^ CD8α^+^ Clec9A^+^ and CD11c^+^ CD11b^+^ CD8α^−^ Clec9A^−^ commonly referred to as CD8 DC and CD11b cDC respectively. Both CD8 and CD11b cDC arise from a common precursor characterized by expression of the C-type lectin receptor DNGR-1, but it appears that pDC arise from a distinct precursor as yet to be defined (10, 11). Recently the CD11b cDC population has been further subdivided on the basis of endothelial cell selective adhesion molecule (ESAM) expression (12). Similar populations can be identified in human such that CD11c^+^ Clec9A^+^ BDCA-3^+^ DCs correspond to CD8 cDC and CD11c^+^ CD11b^+^ BDCA-1^+^ DCs are analogous to CD11b cDC. These populations mainly reside in the secondary lymphoid organs and are present during steady state. Similar populations exist in peripheral tissues but they express a slightly different set of markers (7). CD11c^+^ CD11b^−^ CD103^+^ DC and CD11c^+^ CD11b^+^ CD103^+^ DC in the periphery are equivalent to the CD8 cDC and CD11b cDC populations respectively (13).

### Functional dichotomy in cDC subsets

The two cDC populations also have distinct functions with respect to pathogen recognition, cytokine production, and T cell activation. CD8 and CD103 cDCs express a unique pattern of TLR and other pathogen recognition receptors: they are the only subset to express TLR3 and TLR11 and also uniquely express the C-type lectin Clec9A, DEC205 (CD205), and langerin (CD207) (7, 9). CD8 and CD103 cDC are specialized in the cross-presentation of externally derived antigen on MHC class I for the activation of naïve CD8 T cells. The recent advances in the molecular mechanisms of cross-presentation are covered in a review included in this Research Topic (14). In addition, CD8 cDC appear to preferentially activate CD8 T cells due to the fact that they produce high levels of the cytokines interleukin (IL)-12 and IL-15 that are important for the differentiation and survival of CD8 T cells (15–17). CD8 and CD103 cDCs are the main producers of IL-12, an important cytokine in the differentiation of inflammatory Th1 cells (15, 16, 18). There are also reports suggesting a role for CD8 and CD103 cDCs in the maintenance of peripheral self-tolerance through the deletion of self-reactive T cells (19, 20) and the induction of Treg cells (21). While CD11b cDC are capable of cross-presentation and CD8 T cell activation they appear to be most effective in antigen presentation and activation of CD4 T cells (22) In addition the dermal and intestinal CD11b^+^ CD103^+^ DCs are strong inducers of Treg due to their expression of aldehyde dehydrogenase, the enzyme that converts Vitamin A to retinoic acid, necessary for Treg conversion (23, 24). Several comprehensive reviews on DC subsets and their development have recently been published and can be consulted for further details (7, 9, 13).

### DC and the maintenance of self-tolerance

T and B cell tolerance to self-antigens is acquired during development through clonal deletion of self-reactive cells. DC play an important role in the process of central tolerance in the thymus as they present an array of self-antigens to developing T cells (25). If the T cells have a high affinity for self-antigen they undergo apoptosis and are deleted. Those T cells with a moderate affinity for self are induced to differentiate into forkhead transcription factor (Foxp3)^+^ suppressor cells (26) known as thymus (t)Treg (27). In contrast, peripheral (p)Treg cells arise in the periphery upon Ag contact and several subsets of pTreg can be induced depending on the signals they encounter (28). These include IL-10 (29), TGF-β (30), retinoic acid or vitamin D3 (31, 32), low Ag dose (33), and specific DC subsets (21, 34, 35). As the study of DC biology has evolved, it has become apparent that it is possible to induce the differentiation of Treg using DCs conditioned by immunomodulatory agents (36–39). For example, the presence of TGF-β during the primary stimulation induces the production and expansion of Foxp3^+^ Treg (35). In addition it has been possible to induce IL-10-producing T cells with DC generated using a combination of GM-CSF, TNF-α, and IL-10 (39). Studies have shown that DC producing IDO induce the generation of Treg from naïve T cells (40). Other reports have suggested that immature DC induce the development of functional suppressor cells (36) and that immature DC might, therefore, be therapeutically useful in autoimmunity (37, 38). Recent studies using both nominal and self-Ags have demonstrated that targeting Ags to CD8 cDC in the LN or spleen in the absence of an inflammatory stimulus results in the generation of Treg (41, 42). Similar results have been obtained when Ags were targeted to CD8^−^ DCIR2^+^ DC (21). The CD11b cDC subset has been shown to induce Treg proliferation and expansion in the presence of GM-CSF (43). A recent paper has described a feedback loop in which the number of Tregs in the periphery can be manipulated by altering the relative number of DCs suggesting that DCs play an important role in regulating pTreg numbers (44). We have recently suggested that DC in the periphery may function to maintain tolerance by presenting endogenous self-antigens to naïve T cells thereby inducing pTreg (45).

## The Role of Dendritic Cells in the Pathogenesis/Prevention of T1D

### DC development is defective in T1D

Early studies of NOD mouse revealed abnormalities in the development of myeloid cells demonstrating a reduced proliferative capacity of the bone marrow in response to GM-CSF and M-CSF (46). Several groups, including our own, have identified defects in the development of specific DC subsets in the spleen and other lymphoid organs (47–49). These defects result in a decrease in the numbers of the CD8 cDC population (50, 51), a population which has been shown to have regulatory function (52–54), with a concomitant increase in the CD11b cDC population. Recent studies have also shown that treatment of NOD with Flt 3 ligand (FL) protects mice from diabetes development and this is correlated with an enhancement in the number of CD8 cDC (55, 56). Our own studies demonstrated that treatment of NOD mice with FL increased the number of CD8 cDC and that transfer of these cells to prediabetic NOD mice could have a partial effect in preventing diabetes (50). However a recent study suggested that the timing of FL treatment was critical since disease would be exacerbated if autoimmune CD8 T cells were already present (57).

Several studies have examined the function of bone marrow-derived and isolated DC populations with respect to cytokine production and T cell differentiation and the results have not been consistent. It has been reported by some investigators that NOD DC produce higher levels of the Th1-driving cytokine IL-12 (58–60) while others failed to find such an association (49, 50, 56). Macrophages also appear to be an important source of IL-12 in NOD mice (51, 61). Adenosine plays an important role in modulating the immune response to tissue inflammation (62, 63) and a recent study found that NOD DC express higher levels of adenosine deaminase (ADA), the enzyme responsible for catabolic degradation of adenosine, and that transfer of ADA deficient DC to NOD mice protected them from diabetes (64). These studies suggest that NOD mice have imbalances in DC subsets and alterations in DC function that may contribute to pathogenesis of T1D.

### DC and the maintenance of tolerance in T1D

Dendritic cells are important in both central and peripheral tolerance through the deletion of self-reactive cells and the induction of Treg. NOD mice have defects in central and peripheral tolerance mechanisms (65, 66). Recent studies have shown that the number and/or function of Treg cells decrease as NOD mice age and this is associated with onset of diabetes (67). The development and maintenance of Treg in NOD mice is highly dependent on the presence of co-stimulatory molecules CD80 and CD86 (68). In addition it was recently shown that interaction between the inhibitory molecules PD-1 on T cells and PDL-1 on DC is necessary for the maintenance of tolerance in NOD mice (69). Blockade of this interaction resulted in increased DC/T cell interaction time in the islets and T cell activation leading to the generation of autoreactive effector cells (69). Another study came to the same conclusion by using different antigen constructs to increase the time of antigen presentation (70). IL-2 and signaling via IL-2R are also critical for Treg development and maintenance (71). Recent studies have identified defects in IL-2R signaling in T1D patients (72) and the diabetes susceptibility locus, Idd3, which contains IL-2 was recently shown to control Treg function through an effect on APC (73–75). Several early reports demonstrated that the inflammatory cytokine TNF-α plays an important role in the initiation of T1D (76) and more recently this has been attributed to effects of TNF-α on DC subsets (77). In this study administration of TNF-α to NOD mice was shown to decrease the number of CD8 DC, increase the CD11b DC population, and the DC had a more mature phenotype and activated islet-specific T cells (77) A recent study describing the depletion of Treg in NOD mice showed that the increase in diabetes in these animals was associated with aggressive infiltration of pancreatic islets by DC rather than CD4 T cells (78). These results suggest that tTreg in this context may prevent autoimmunity by controlling the migration of cDC into the islet.

### DC contribute to the development of T1D

Analysis of the phenotype and function of BM-derived DC in NOD mice have suggested that these cells produce higher levels of IL-12p70, and that this related to increased expression of NFκB (58). Our own studies suggest that BM-derived DC from NOD can produce higher or lower levels of IL-12p70 depending on the culture conditions and activation stimuli used (49, 79, 80). Recent studies using DTR transgenic mice that allow the targeted depletion of macrophages or DC have demonstrated that the CD11b cDC population is responsible for presenting antigen to autoreactive T cells (81). Ablation of these cells protected NOD mice from diabetes development. The interaction of iNKT cells with cDC was shown to lead to either protection via induction of Treg or exacerbation of disease if the interaction occurred at the same time as TLR4 ligation (82).

The role of pDC in diabetes pathogenesis is somewhat more controversial and there are conflicting reports in the literature. Depletion of pDC using the CD11c-DTR system caused accelerated disease (81), suggesting a protective role for pDC. The protection induced by pDC was correlated with increased local production of IDO and increased numbers of NKT cells in the pancreas. In a model of virus infection it was shown that activation of iNKT cells stimulated TGF-β production from pDC, which led to the induction of Treg and protection from diabetes (83). In contrast, another study found that IFN-α could be detected in the pancreas of NOD mice at early ages and that blocking the ability of mice to respond to IFN-α prevented diabetes (84). Furthermore these investigators showed that depletion of pDC using a depleting antibody reduced the incidence of diabetes (85). Another study revealed a crosstalk between B-1a B cells, neutrophils and pDC that contributed to the induction of diabetes via type 1 IFN production (86). This study suggests that DNA from dying β cells together with antibodies from B-1a B cells form complexes that are potentiated by neutrophils and lead to the induction of type 1 IFN by pDC (86). The authors show that this is seen only in NOD mice and speculate that this is related to the defects in the ability of NOD macrophages to clear debris (87). Several studies have examined the role of pDC, type 1 IFN and viral infection in the induction of T1D; reviewed in Swiecki et al. (88). The situation is complex with some studies showing exacerbation of diabetes (89, 90) with viral infection and others showing protection (91, 92). It has been suggested that whether viral infection leads to diabetes induction is related to the tropism of the virus and the local environment in which type 1 IFN is produced (88). Thus a virus infecting the pancreas may induce local type 1 IFN, which protects islets from infection and damage, whereas in other situations the type 1 IFN, produced by pDC following viral infection, could result in activation of autoreactive T cells and diabetes exacerbation (88). It has been reported that pDC also accumulate in the islets of NOD mice (93) at a later time point, around 10 weeks of age. The infiltrating pDC were shown to express IDO, which is characteristic of a more tolerogenic phenotype of DC (94) leading to the speculation that these cells may be attempting to control ongoing T cell activation. Studies in the next few years will hopefully clarify these contrasting results on the role that pDC play in diabetes pathogenesis.

A novel population of cDC, termed merocytic (mc)DC, have recently been identified and shown to be responsible for cross-presentation of islet antigens to CD8 T cells and direct presentation to CD4 T cells (95, 96). The mcDC express CD11c but are negative for both CD11b and CD8, they accumulate in the spleen as NOD mice age and were shown to secrete large amounts of type 1 IFN (95, 96). In addition transfer of mcDC pulsed with irradiated islets to non-diabetic NOD mice accelerated the onset of diabetes (95, 96). The number of mcDC was recently mapped to the *Idd13* congenic interval suggesting that the relative number of these cells is genetically determined (97). Further studies are required to determine the significance of this DC population, and whether a counterpart of these cells exists in human.

Dendritic cells are constitutively present within islets of normal mice and have been shown to express peptide-MHC (pMHC) complexes containing peptides derived from islet antigens (98). Two major DC subsets can be found within islets; the CD11b cDC and CD103 cDC (99, 100). CD103 DC depend on FL for their homeostasis whereas islet CD11b DC appear to arise from monocytes and are unaffected by the absence of FL (101). The number of DCs in the islets remains relatively stable but these numbers increase following T cell infiltration and inflammation (98, 99, 102). In addition the phenotype of the DC becomes more inflammatory with increases in the expression of co-stimulatory molecules and MHC (2). The evidence suggests that the initiation of the autoimmune response occurs within the draining pancreatic lymph node (PLN) since removal of the PLN prevents diabetes (103) and several studies have shown that the initial proliferation of islet-specific CD4 and CD8 T cells takes place in the PLN (104, 105). Islet CD103 DC migrate to the PLN where they present islet antigens to specific CD4 and CD8 T cells (2, 101), and when T cells infiltrate the islet they localize to DC-rich areas (2). The movement of DC and T cells within the islet can now be visualized using a novel two-photon imaging technique (106). In addition pancreatic CD103 DC from NOD mice have been shown to express less IL-10 than similar populations from non-diabetic strains suggesting that these have lost their ability to induce tolerance (107). Islet CD11b DC are relatively poor at presenting antigen under steady state conditions but they accumulate as inflammation increases and become more mature. Since CD11b DC do not appear to migrate to PLN their role appears to be in the modulating the local tissue response (101).

### DC in human T1D

Several studies have examined the blood of newly diagnosed T1D patients for the presence of DC subsets. pDCs have been shown to be increased (108) or decreased (109) at the time of diagnosis. A recent detailed longitudinal analysis of immune parameters in newly diagnosed T1D children has revealed that reduced numbers of cDC1s and NKT cells at the time of diagnosis are correlated with reduced residual β cell function 1 year later (110). Another study found decreased numbers of cDCs and pDCs in newly diagnosed pediatric T1D patients and also observed a decrease in the expression of CCR2 on these cells (111). Other studies have found correlations with vitamin D levels and immune cells in T1D patients (112). A study of pancreatic biopsies on a small number of new onset T1D patients revealed the presence of infiltrating macrophages and DC that produce TNF-α (113). A recent description of three cases of fulminant T1D secondary to enterovirus infection revealed marked islet infiltration of activated DC and macrophages and the presence of inflammatory cytokines (114). Thus it is likely that DCs will be shown to contribute to the onset of human T1D.

## Dendritic Cells as Therapy for T1D

The fact that DCs play an important role in the induction and maintenance of self-tolerance has made them attractive targets for therapeutic interventions. Three main strategies have been employed and these include the adoptive transfer of specific DC subsets, the *in vitro* expansion of Tregs with specific DCs and the *in vivo* targeting of DC subsets (115). As discussed above the complexity of DC subsets and the plasticity of their function have made this a challenging objective, since there is a fine balance between the immunostimulatory and immunoregulatory functions of DC (45, 116, 117). DCs with a so called semi-mature phenotype, which consists of increased MHC and co-stimulatory molecule expression but low inflammatory cytokine production are thought to be most effective in inducing tolerance in the context of autoimmunity (118, 119). This maturation state can be induced by exposing DC to the cytokine TNF-α and DCs treated in this way have been shown to prevent experimental allergic encephalomyelitis (EAE) (118). In general the function of these DCs is to induce the expansion of Treg or other regulatory mechanisms such as Th2 cells. The attraction for using DCs as therapeutic agents is that since they present specific antigen to T cells it is possible to envision the generation of antigen-specific tolerance (120).

### Adoptive transfer of therapeutic DC

Dendritic cells were first used as therapeutic agents to prevent diabetes in NOD mice over 20 years ago by Clare-Salzler et al. (121). In this study DCs isolated from the PLN of NOD mice and transferred to prediabetic NOD mice were able to prevent disease onset whereas DCs from other LN or spleen were not effective. Later studies by the same group have suggested that DC in the PLN are more mature and that this is important for their therapeutic effect (122). We have previously shown that the injection of semi-mature bone marrow-derived DC prior to the onset of destructive insulitis prevents the subsequent development of diabetes (123, 124). The therapeutic DC populations expressed high levels of co-stimulatory molecules (CD80, CD86, and CD40) and produced low levels of IL-12p70 following CD40 ligation (49). Further investigation revealed that the therapeutic DC population changed the cytokine milieu in treated NOD mice. Whereas NOD mice generally exhibit a strong bias toward type 1 cytokine production, injection of DC induced the production of type 2 cytokines (125). In addition we (124) and others (126, 127) showed that injection of DC transduced with the IL-4 gene could effectively prevent diabetes in NOD mice when given at later time points, past the onset of invasive insulitis. We also confirmed, using *in vitro* studies, that therapeutic DC could drive the differentiation of Th2 cells (79), whereas non-therapeutic DC did not. The therapeutic DCs were not treated with any tolerogenic agents and did not require the addition of islet-derived peptides but the cultures were performed in FBS, which was subsequently shown to induce a Th2 environment that may have been non-specifically contributing to the therapeutic effect (128). In more recent experiments we have shown that the therapeutic DC population induces the expansion of Treg in the presence of low dose antigen (129) and DCs cultured in the absence of FBS are equally able to induce Treg expansion (unpublished observations). In addition it has been shown that DC grown in autologous serum were able to prevent diabetes but only when pulsed with insulin-derived peptides (130).

In order to ensure that DCs maintain a tolerogenic phenotype and drive tolerance rather than immunity several approaches aimed at conditioning the DC have been explored. These have included treating DC with cytokines such as IL-10 (130, 131), IL-10/TGF-β (132), and TSLP (IL-25) (133), pharmacological agents such as dexamethasone and vitamin D_3_ (134), carbon monoxide (CO) (135), anti-CTLA-4 antibody (136), secretory IgA (137) among others. In general, the treatment of DCs with these agents induces a semi-mature phenotype in the DC characterized by reduced expression of co-stimulatory molecules, reduced inflammatory cytokine production and resistance to further maturation stimuli. The infusion of these modified DC into NOD mice results in the deletion of autoreactive CD4 T cells and the generation of islet-specific Treg (130, 131, 133, 136, 137). One exception to this was the treatment of NOD mice with CO-treated DC, which reduced β1-integrin expression by CD8 T cells and thus inhibited their migration into the islet (135). Another approach has been the use of anti-sense oligonucleotides to down-regulate the expression of the co-stimulatory molecules, CD40, CD80, and CD86 on DC (138). This approach prevented diabetes in NOD mice through the induction of Treg and a phase 1 clinical trial using this approach was initiated (139).

### DC used to expand regulatory T cells *in vitro*

Dendritic cells have also been used *in vitro* in order to expand Treg cells, and these Treg can be adoptively transferred into individuals with autoimmunity with the hope of curbing or arresting the inflammatory response. An early study expanded Treg, isolated from an islet-specific TCR transgenic NOD mice (BDC2.5), with peptide pulsed DC and IL-2 and showed that these cells could prevent diabetes in prediabetic NOD mice (34). Further studies from this group indicated that DC expressing higher levels of the co-stimulatory molecule, CD86 were the most efficient at inducing Treg expansion (140). The CD11b cDC subset has been shown to induce Treg proliferation and expansion in the presence of GM-CSF (43). A recent study examining the mechanism by which GM-CSF treatment prevents diabetes has further suggested that the expression of OX40L and the Notch3 ligand Jagged1 on DC was necessary for Treg expansion *in vitro*, although this study did not determine whether these polyclonal expanded Treg were effective in preventing diabetes (141). Some of the approaches to generate tolerogenic DC are being developed for clinical trial in the context of transplantation (142) but these are not yet being fully developed for use in autoimmune disease. A recent study compared the ability of several DC conditioning regimens to induce suppressive Treg in the human system (143). This study found that DC pretreated with IL-10 induced the most suppressive Treg, while TGF-β, rapamycin, and dexamethasone were less effective (143).

### *In vivo* targeting of DC

In general these types of therapies have involved the use of reagents that deliberately target specific DC subsets but these can also include approaches that, while not directly targeting DC, act by changing the local DC environment. Recent studies using both nominal and self-antigens have demonstrated that targeting antigens to CD8 cDC in the LN or spleen using anti-CD205 antibodies coupled to specific antigens in the absence of an inflammatory stimulus results in the generation of Treg (41, 42). Similar results have been obtained when antigens were targeted to CD11b cDC (21). A recent study used anti-CD205 antibodies coupled to the peptide mimetope for the CD4 BDC2.5 T cell to target DC in NOD mice (144). This resulted in the generation of long-lived and stable BDC2.5 Treg but this had no impact on the development of diabetes. However when the anti-CD205 was coupled to the proinsulin protein diabetes was prevented (144). Similar studies using an anti-CD205 coupled to HA and given to INS-HA/TCR-HA mice also demonstrated efficacy in the prevention of diabetes (145). In this mouse model the foreign antigen HA is expressed in the pancreas under the control of the rat insulin promoter and the T cells are all transgenic for an HA-specific TCR (145). These results suggest that the choice of antigen for targeting will be critical if this approach is to move forward. A similar approach has been used to induce tolerance in islet-specific CD8 T cells. These studies reported initial activation of adoptively transferred islet-specific CD8 cells followed by deletion of these cells, but the impact of these on diabetes development was not reported (146).

It has been known for some time that varying the dose of the stimulating antigen has a profound effect on T cell differentiation (147–149). Interest in this area has been renewed by the observation that the induction of Treg is optimal in situations of low TCR signal strength (33, 129, 150). The induction of Treg by low dose antigen occurs optimally when DC are presenting the antigen (129), and while the addition of TGF-β is not necessary a recent study showed that TGF-β production by T cells is required (151). Interestingly the most effective induction of Treg *in vivo* occurs when low doses of a high affinity peptide are used, rather than higher doses of low affinity peptides (152). Furthermore it was recently shown that the affinity of the peptide affects the duration of DC/T cell contacts *in vivo* providing an explanation for why low dose of more potent pMHC complexes are most effective at driving Treg expansion *in vivo* (153). Several approaches have been attempted to deliver low dose antigen *in vivo* including the continuous delivery of low dose antigen via osmotic pump (154) and the direct targeting of DC subsets as discussed above (21, 41, 42). In the context of T1D a recent study demonstrated that low doses of a high affinity insulin peptide mimetope was more effective at preventing diabetes than the native peptide (155). These results suggest that long-lasting peripheral tolerance could be achieved by delivering the appropriate dose of antigen to steady state DC in the periphery. The challenge will be translating this to the human situation although a recent paper showed that human insulin-specific Treg could be induced by low dose stimulation (156).

Another way to target DC *in vivo* is to use microparticles (157). A recent study used microspheres loaded with anti-sense oligonucleotides specific for CD40, CD80, and CD86 and showed prevention of diabetes along with an increase in Treg in treated NOD mice (158). These investigators further showed that repeated treatment with these microspheres could reverse diabetes in newly diagnosed NOD mice. The microspheres were taken up by resident DC in the spleen and these DC showed reduced expression of the co-stimulatory molecules (158).

In a study of combined therapy with oral anti-CD3 and intravenous anti-CD20 antibodies the prevention of diabetes seen in these animals was correlated with an increase in Treg and IL-10-producing T cells. Furthermore a population of IL-27 producing DCs was found to be responsible for the induction of IL-10-producing T cells in this system (159). As discussed above FL treatment of NOD mice in some circumstances prevents T1D (55, 56) but the timing of FL administration has to be before the onset of autoimmunity otherwise disease is exacerbated (57). Treatment of NOD mice with soluble CTLA-4 has been shown to restore the tolerance inducing properties of DC through the IFN-γ dependent activation of IDO. In this model CTLA-A binds to B7 molecules on DC and this stimulates the release of IFN-γ, which activates the immunosuppressive mechanism of tryptophan catabolism (160). A novel approach has been to induce accumulation of thymic DC populations which results in an increase in suppressive Tregs. A recent study identified the delta-like ligand 4 (Dll4)-Notch signaling pathway as important in controlling the number of thymic DCs (161). These investigators found that blockade of the Dll4-Notch pathway resulted in an increase in thymic DC numbers and thymic Treg. Treatment of NOD mice with these blockers not only prevented the establishment of disease in prediabetic animals but also was able to reverse disease in newly diagnosed diabetic animals (161). This protection was dependent on the presence of Treg since treatment of non-diabetic animals with a Treg-depleting antibody reversed the protection (161). Several studies have reported the use of α-GalCer, an agonist for iNKT cells, as a means to prevent disease in NOD mice (162–164). Recent studies have suggested that α-GalCer treatment induces tolerogenic DC populations that induce non-inflammatory islet-specific T cells (165), and novel derivatives of α-GalCer have been developed that have the same effect without some of the profound suppressive effects on iNKT function (166).

## Clinical Trials in T1D Involving DC

As discussed above a phase I clinical trial utilizing autologous monocyte-derived DC treated with anti-sense oligonucleotides to down-modulate co-stimulatory molecules has been conducted. In this trial of 10 patients with established T1D, 7 were given the immunosuppressive DC and 3 were given unmanipulated DC. In this trial no adverse events were observed and there were few changes noted in terms of insulin requirements, immune cell phenotype, and cytokine production (139). Interestingly, further analysis of this clinical trial revealed an increase in certain B cell populations and subsequent *in vitro* studies revealed that human DC treated with these anti-sense oligonucleotides could induce the proliferation of immunosuppressive B cells (167). A second trial using this same approach is now ongoing but results are not yet available. The field of DC-based vaccines is much more advanced in the setting of cancer and many clinical trials have been conducted; reviewed in the current Research Topic (Butterfileld, under review). Many features of cancer DC vaccines have been studied and it has become apparent that characteristics such as the form of the antigen used to pulse the DC, the conditioning regimen of the DC, and the route of administration all play critical roles in determining the outcome of the DC-based immunization. These features will need to be taken into consideration as more DC-based therapies are proposed for the treatment of T1D. There have been numerous clinical trials in T1D with the aim of inducing antigen-specific tolerance (168). These trials utilize islet-derived antigens, such as insulin GAD65 or hsp 70, and various routes of administration have been tested including oral, nasal, and intradermal. So far these trials have failed to have a significant impact on disease although in some cases evidence for immune tolerance was observed (168). It is to be expected that the success of such trials will depend on the APC, most likely a DC, targeted by these antigen formulations. At the present time not much attention is being paid to the nature of the DC presenting the antigen in these trials but, in view of our increasing understanding of the complexity of DC phenotype and function this is likely to change.

## Future Perspectives

As discussed at the beginning of this review many of the studies that examine the therapeutic potential of DC in the treatment/prevention of T1D have been performed in the NOD mouse. This has been a very useful model for identifying susceptibility genes and understanding the progression to disease but therapeutic approaches that have successful in this mouse model have not translated well to the clinic (169). DCs play a pivotal role in setting the tone of the immune response. On the one hand they contribute to the development and maintenance of self-tolerance and on the other they contribute to the breaking of that tolerance and the initiation of disease. A deeper understanding of how DC can influence the development of autoimmune diabetes will aid in the development of novel therapeutic strategies. A great deal of progress has been achieved in the last several years and we have a better understanding of how DC can both maintain and break self-tolerance. The challenge in the future will be to use this knowledge to achieve the ultimate goal of inducing antigen-specific tolerance to prevent autoimmunity without causing widespread immune suppression.

## Conflict of Interest Statement

The author, editor and chief editor declare that while the author Penelope Morel and the editor Lisa Butterfield are currently employed by the same institution, University of Pittsburgh, USA, there has been no conflict of interest during the review and handling of this manuscript, and the manuscript reviewers involved were from other institutions.
